# Electrophysiological effects of rosiglitazone on heart ventricular papillary muscles of control and diabetic histidine decarboxylase knock-out and wild-type mice

**DOI:** 10.1186/1471-2210-11-S2-A53

**Published:** 2011-09-05

**Authors:** Andrea Szebeni, Ágnes Kovács, Valéria Kecskeméti

**Affiliations:** 1 Department of Pharmacology and Pharmacotherapy, Semmelweis University, 1089 Budapest, Hungary

## Background

Rosiglitazone is a thiazolidinedione derivative oral hypoglycemic agent active in both diabetic animal models and type 2 diabetic patients. Rosiglitazone is a high affinity ligand for the peroxisome proliferator-activated receptor gamma, which is responsible for the insulin-sensitizing action of the compound. Recent large clinical trials found an association between the antidiabetic drug rosiglitazone therapy and increased risk of cardiovascular adverse events.

## Methods

The aim of this report is to elucidate the cardiac electrophysiological properties of rosiglitazone on control and diabetic murine ventricular papillary muscles using conventional microelectrode technique.

## Results

In control histidine-decarboxylase knock-out mice (HDC-KO) as well as in their wild-types (WT) rosiglitazone (1–30 μM) shortened AP duration at the 90% level of repolarization (APD_90_) and increased the AP amplitude (APA) in a concentration-dependent manner. Moreover, rosiglitazone reduced the maximum velocity of depolarization (V_max_). In diabetic animals we detected very similar effects.

## Conclusions

The action potential changes caused by rosiglitazone probably can be explained by ion channel effects. The observed alterations may carry a serious proarrhythmic risk in case of overdose intoxication with rosiglitazone, especially in patients having multiple cardiovascular risk factors, like elderly diabetic patients.

